# Post‐Endoscopic Retrograde Cholangiopancreatography Bleeding After Endoscopic Stone Removal in Dialysis Patients: A Multicenter Retrospective Study

**DOI:** 10.1002/deo2.70395

**Published:** 2026-08-07

**Authors:** Masaki Nishimura, Haruo Miwa, Takashi Kaneko, Endo Kazuki, Oishi Ritsuko, Suzuki Yuichi, Tsuchiya Hiromi, Suzuki Yoshimasa, Yoshihiro Goda, Akihiro Funaoka, Nobutaka Doba, Kuniyasu Irie, Yuki Miura, Tomohiro Ishii, Masaaki Kondo, Kazuya Sugimori, Shin Maeda

**Affiliations:** ^1^ Gastroenterological Center Yokohama City University Medical Center Kanagawa Japan; ^2^ Department of Gastroenterology Fujisawa City Hospital Kanagawa Japan; ^3^ Department of Gastroenterology Yokohama City University Hospital Kanagawa Japan; ^4^ Department of Gastroenterology Yokosuka City Hospital Kanagawa Japan; ^5^ Department of Gastroenterology Japanese Red Cross Hadano Hospital Kanagawa Japan; ^6^ Department of Gastroenterology Saiseikai Yokohamashi Nanbu Hospital Kanagawa Japan; ^7^ Department of Gastroenterology Yokohama Minami Kyosai Hospital Kanagawa Japan; ^8^ Department of Gastroenterology Yokohama City University Graduate School of Medicine Kanagawa Japan

**Keywords:** dialysis, endoscopic retrograde cholangiopancreatography, hemorrhage, pancreatitis, postoperative complications

## Abstract

**Objectives:**

Post‐endoscopic retrograde cholangiopancreatography (post‐ERCP) bleeding occurs more frequently in dialysis patients than in non‐dialysis patients. This study aimed to identify risk factors for post‐ERCP bleeding following stone removal in patients undergoing dialysis.

**Methods:**

This multicenter retrospective study enrolled 102 consecutive dialysis patients who underwent endoscopic stone removal at seven institutions between January 2009 and March 2022. Baseline characteristics, procedural details, stone removal outcomes, adverse events (AEs), and risk factors for post‐ERCP bleeding were analyzed.

**Results:**

Of the 102 patients, 65 were men. Median patient age was 77 years (range, 51–91 years). Antithrombotic drugs were administered to 64 patients (63%). Endoscopic sphincterotomy, endoscopic papillary balloon dilation, and endoscopic papillary large balloon dilation were performed in 58 (57%), 20 (20%), and 11 (11%) patients, respectively. AEs occurred in 23 patients (23%), including bleeding in eight patients (8%), acute pancreatitis in nine patients (9%), cholangitis in seven patients (7%), and others in two patients (2%). Multivariate analysis identified intraoperative bleeding and continued aspirin use as independent risk factors for post‐ERCP bleeding. Similar associations were observed in subgroup analyses restricted to patients with a naïve papilla.

**Conclusions:**

Intraoperative bleeding and continued aspirin use were associated with post‐ERCP bleeding following stone removal in dialysis patients, highlighting the importance of careful periprocedural hemostatic management in this population.

**Trial Registration:**

Not applicable.

## 1 Introduction

Recently, with the increasing prevalence of chronic kidney disease, the number of dialysis patients with gallstone‐related disease is increasing [1], thereby creating an expanding demand for the endoscopic management of common bile duct stones.

Endoscopic retrograde cholangiopancreatography (ERCP) is the first‐line treatment for common bile duct stones, typically involving papillary procedures such as endoscopic sphincterotomy (EST) or endoscopic papillary balloon dilation (EPBD) prior to stone removal [2]. Endoscopic stone removal has demonstrated high technical success rates and low rates of adverse events (AEs) in the general population [3].

Although endoscopic stone removal in dialysis patients has comparable technical success to that in non‐dialysis patients [4], safety remains a concern owing to comorbidities such as cardiovascular disease, cerebrovascular disease, diabetes mellitus, and arteriosclerosis [5, 6, 7]. Several studies have reported that ERCP‐related AEs may occur more frequently in patients undergoing dialysis than in the general population [8, 9].

Among these AEs, post‐ERCP bleeding is of particular concern because dialysis patients are predisposed to bleeding [10, 11, 12, 13] owing to platelet dysfunction, reduced coagulability, vascular fragility, and the use of anticoagulants during hemodialysis [14].

In the treatment of common bile duct stones, papillary procedures such as EST and EPBD are necessary to safely extract the stones. Particularly, repeated stone extraction maneuvers through the papilla may cause mechanical trauma and increase the risk of bleeding. Therefore, careful periprocedural management of bleeding is necessary when treating dialysis patients.

Previous studies have identified several factors associated with post‐ERCP bleeding in patients undergoing dialysis [15, 16, 17]. Although several reports have revealed substantial associations between antithrombotic agents and ERCP‐related procedures [15, 18], some studies have contrarily reported that antiplatelet agents do not increase the risk of post‐ERCP bleeding [16]. Currently, perioperative management of antithrombotic agents does not differ between dialysis and non‐dialysis patients. However, because dialysis patients are at an increased risk of bleeding, the safety of continuing antiplatelet agents during ERCP remains unclear. Antithrombotic therapies are increasingly used to reduce thromboembolic events in patients undergoing dialysis; however, their use may increase the risk of procedural bleeding. Indeed, numerous studies have indicated that intraoperative bleeding is a strong, independent risk factor for post‐ERCP bleeding [13, 18].

This study aimed to evaluate the clinical outcomes of endoscopic bile duct stone removal in dialysis patients and to identify risk factors for post‐ERCP bleeding, with a particular focus on perioperative antithrombotic management.

## 2 Methods

### 2.1 Patients

This multicenter retrospective study enrolled 102 consecutive patients aged >20 years who were undergoing dialysis and underwent endoscopic stone removal at seven institutions between January 2009 and March 2022.

### 2.2 Data Extraction

Data were collected on the study design, patient characteristics, periprocedural antithrombotic management, procedural details, technical success, and AEs.

### 2.3 Treatment Indication

Common bile duct stones were diagnosed based on imaging findings. ERCP was recommended for symptomatic patients and selectively performed in asymptomatic patients after informed consent. Cholangitis was diagnosed according to the Tokyo Guidelines 2018 [19].

### 2.4 ERCP Procedure

All ERCP procedures were performed or supervised by experienced endoscopists using a side‐viewing duodenoscope. Papillary interventions, including EST and EPBD, or endoscopic papillary large balloon dilation (EPLBD), were selected based on stone size, anatomical factors, procedural difficulty, and endoscopist discretion. In general, EST alone was preferred for patients with one or two stones measuring ≤10 mm, whereas EST combined with EPLBD was considered for larger stones, multiple stones, distal bile duct dilation, or anatomically difficult cases such as periampullary diverticula. In patients receiving antithrombotic therapy or considered to be at high risk of bleeding, EPBD‐based approaches were preferentially selected when clinically feasible. EST was performed concurrently with EPLBD; a medium incision was used for EST alone, whereas a small incision was used for EST combined with EPLBD. After selective biliary cannulation, cholangiography was performed to confirm the presence of choledocholithiasis, and patients without stones were excluded. Stone removal was attempted using standard techniques, including retrieval baskets or balloon catheters, and mechanical lithotripsy was performed when necessary. Successful stone removal was defined as the absence of residual stones on the final cholangiogram. If residual stones remained, endoscopic biliary drainage was performed.

### 2.5 Anticoagulation in Dialysis

Dialysis and anticoagulation during dialysis were managed according to institutional practice and individual bleeding risk.

### 2.6 Antithrombotic Drug

The perioperative management of antithrombotic medications followed the guidelines for gastrointestinal endoscopic procedures in patients receiving antithrombotic therapy [20]. In patients expected to require high‐bleeding‐risk papillary interventions such as EST, temporary discontinuation of antithrombotic agents associated with a high risk of bleeding, except for aspirin, was generally considered according to the guidelines. Aspirin was generally continued, although it was discontinued in selected patients considered to be at low thromboembolic risk. In patients considered to be at high thromboembolic risk, continuation of antithrombotic therapy and selection of lower‐bleeding‐risk procedures, such as EPBD‐based approaches, were preferentially considered when clinically feasible. In urgent ERCP cases requiring stone extraction, procedures were occasionally performed without sufficient withdrawal periods for antithrombotic agents. Final decisions regarding antithrombotic management and procedural strategy were made at the discretion of the attending physicians and endoscopists.

### 2.7 Definition of ERCP‐Related AEs

**FIGURE 1:**
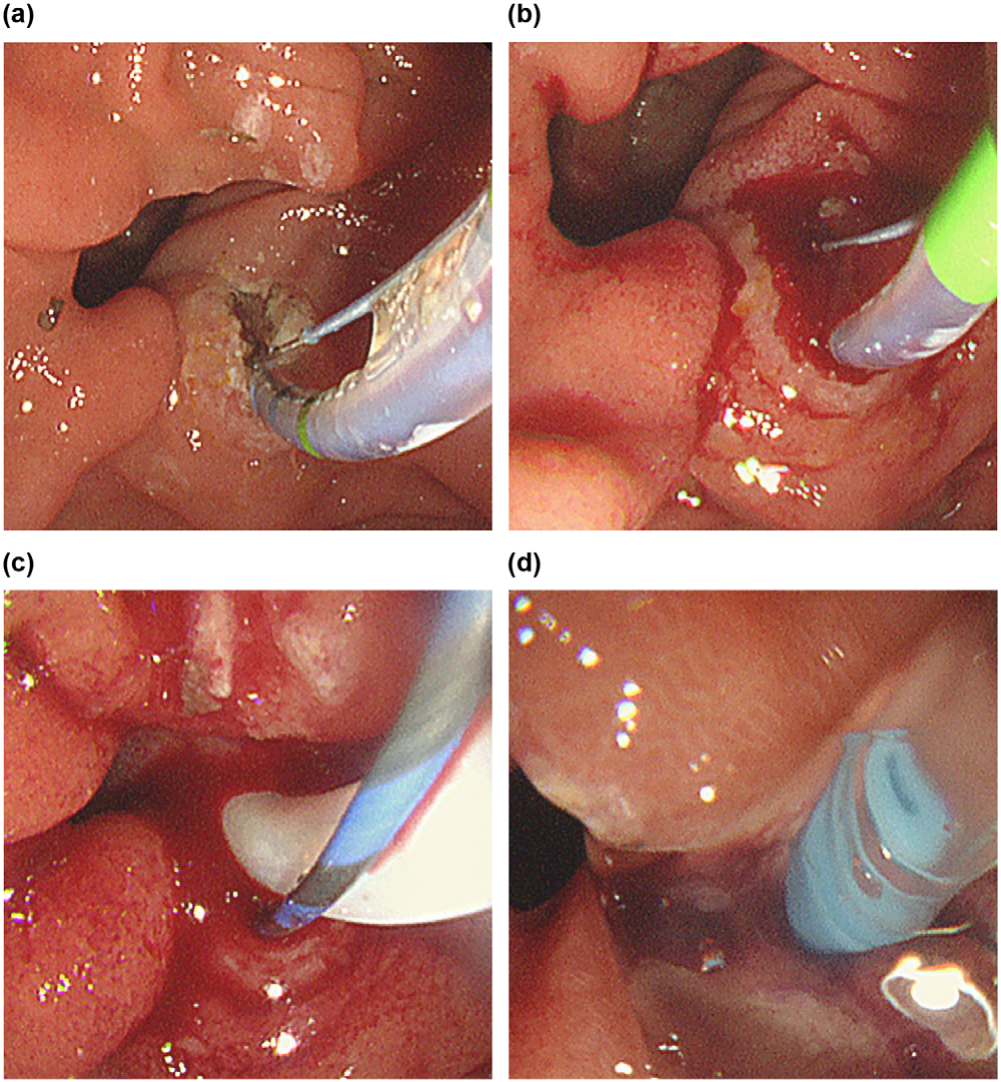
Intraoperative bleeding during endoscopic sphincterotomy (EST) in a dialysis patient. (a) EST is performed after biliary cannulation. (b) Intraoperative bleeding occurs during EST. (c) Hypertonic saline epinephrine is injected at the bleeding point. (d) A self‐assembling hemostatic gel is applied to the bleeding site.

ERCP‐related AEs were defined and graded according to the ASGE Lexicon [21]. ERCP‐related bleeding was classified as intraoperative or post‐ERCP bleeding. Intraoperative bleeding was defined as persistent bleeding documented by the endoscopist as requiring continued endoscopic observation or hemostatic intervention during the procedure (Figure 1), whereas post‐ERCP bleeding was defined as bleeding occurring within 5 days after the procedure. Bleeding was diagnosed based on clinical evidence of melena or hematemesis, accompanied by a decrease in hemoglobin levels of ≥2 g/dL. The severity of bleeding AEs was graded as mild, moderate, and severe according to hospitalization duration (1–3, 4–9, and >10 days, respectively), the need for surgery or intensive care, or procedure‐related death. Post‐ERCP pancreatitis was defined as new‐onset abdominal pain accompanied by an elevation in pancreatic enzyme levels to ≥3 times the upper limit of normal at 24 h after the procedure. Other AEs not included in the ASGE Lexicon, such as hyperamylasemia or elevated liver enzyme levels, were assessed according to ASGE recommendations.

### 2.8 Subgroup Analysis

A naïve papilla was defined as a major duodenal papilla with no prior endoscopic papillary intervention, including EST, EPBD, or EPLBD. Because previous papillary intervention may influence the need for additional papillary procedures during ERCP, a prespecified subgroup analysis was performed in patients with a naïve papilla.

### 2.9 Statistical Analysis

Statistical analyses were performed using JMP Pro version 12. Continuous variables were compared using appropriate parametric or nonparametric tests, and categorical variables using Fisher's exact test. Univariate logistic regression analyses were conducted to determine factors associated with post‐ERCP bleeding. Variables considered clinically relevant and those selected based on univariate analysis were included in the multivariate model. A two‐sided *p*‐value < 0.05 was considered statistically significant. Subgroup analyses were performed in patients with a naïve papilla.

## 3 Results

### 3.1 Baseline Characteristics

The median age of the patients was 77 years (range, 51–91 years), with 65 men and 37 women. The mean platelet count was 17.8 × 10^4^/µL, and the mean prothrombin time‐international normalized ratio was 1.13. The comorbidities included hypertension (70 patients), heart disease (61 patients), diabetes mellitus (47 patients), cerebrovascular disease (21 patients), and hepatic cirrhosis (three patients). Diabetic nephropathy was the leading cause of dialysis in 44 patients, with a median duration of 60 months (range, 1–456 months). During dialysis, the most commonly used anticoagulant was nafamostat mesylate (57 patients), followed by unfractionated heparin (28 patients) and low‐molecular‐weight heparin (17 patients). Preprocedural cholangitis was present in 35 patients (34%) at the time of ERCP (Table 1).

**TABLE 1:** Baseline characteristics of the study participants.

*n*	102
Age (years), Median (range)	77 (51−91)
Sex, Male, *n* (%)	65 (64)
Blood test, Mean ± SD	
Platelets (×10^4^)	17.8 ± 8.6
PT‐INR	1.13 ± 0.33
APTT (sec)	36.5 ± 9.6
Comorbidities, *n* (%)	
Hypertension	70 (69)
Heart disease	61 (60)
Diabetes mellitus	47 (46)
Cerebrovascular disease	21 (21)
Hepatic cirrhosis	3 (2.9)
Dialysis period (months), median (range)	60 (1–456)
Reason for renal disease, *n* (%)	
Diabetic nephropathy	44 (43)
Chronic glomerulonephritis	25 (25)
Nephrosclerosis	16 (16)
Other	17 (17)
Anticoagulants used during dialysis, *n* (%)	
Nafamostat mesylate	57 (56)
Unfractionated heparin	28 (27)
Low‐molecular‐weight heparin	17 (17)
Use of antithrombotic drugs, *n* (%)	64 (63)
A single antithrombotic	49 (48)
Multiple antithrombotic	15 (15)
Continued use of a single antithrombotic	19 (19)
Discontinued use of a single antithrombotic	33 (32)
Discontinued use of multiple antithrombotics	6 (5.9)
Heparin alternative therapy	6 (5.9)
Preprocedural cholangitis, *n* (%)	35 (34)

Abbreviations: APTT, activated partial thromboplastin time; PT‐INR, prothrombin time‐international normalized ratio; SD, standard deviation.

### 3.2 Antithrombotic Drug

A total of 64 patients (63%) were taking antithrombotic drugs at the time of admission, including aspirin, clopidogrel sulfate, cilostazol, dipyridamole, warfarin, and rivaroxaban (Table 1). Moreover, 15 (15%) were using multiple antithrombotics, 49 (48%) were using a single antithrombotic, 33 (32%) discontinued the use of a single antithrombotic, and six patients (6%) discontinued the use of multiple antithrombotic drugs; six patients (6%) underwent heparin alternative therapy. The majority of patients, specifically 37 (36%), were taking aspirin. The second largest group, comprising nine patients (9%), were taking a combination of aspirin and a thienopyridine. Among all patients, 14 (14%) continued the use of aspirin (Table 2).

**TABLE 2:** Details of antithrombotic agents and perioperative management.

	All patients	Continued single antithrombotic drug use	Discontinued antithrombotic use		Heparin
Antithrombotic type	(*n* = 64)	(*n* = 19)	Single (*n* = 33)	Multiple (*n* = 6)	(*n* = 6)
Aspirin	37 (58%)	10 (53%)	27 (82%)	0 (0%)	0 (0%)
Thienopyridines	3 (4.7%)	1 (5.3%)	2 (6.0%)	0 (0%)	0 (0%)
Cilostazol	1 (1.6%)	0 (0%)	1 (3.0%)	0 (0%)	0 (0%)
Dipyridamole	1 (1.6%)	0 (0%)	1 (3.0%)	0 (0%)	0 (0%)
Warfarin	6 (9.4%)	2 (11%)	2 (6.0%)	0 (0%)	2 (33%)
DOAC	1 (1.6%)	1 (5.3%)	0 (0%)	0 (0%)	0 (0%)
Aspirin + thienopyridine ^a^	9 (14%)	4 (21%)	0 (0%)	3 (50%)	2 (33%)
Aspirin + warfarin	2 (3.1%)	0 (0%)	0 (0%)	0 (0%)	2 (33%)
Aspirin + thienopyridine + warfarin	1 (1.6%)	0 (0%)	0 (0%)	1 (17%)	0 (0%)
Thienopyridines + cilostazol	1 (1.6%)	1 (5.3%)	0 (0%)	0 (0%)	0 (0%)
Thienopyridines + cilostazol + warfarin	1 (1.6%)	0 (0%)	0 (0%)	1 (17%)	0 (0%)
Cilostazol + warfarin	1 (1.6%)	0 (0%)	0 (0%)	1 (17%)	0 (0%)

Abbreviation: DOAC, direct oral anticoagulant.

^a^ Continued use of aspirin and discontinued use of thienopyridine.

### 3.3 Outcomes and AEs of ERCP

A total of 89 patients (87%) had naïve papillae. EST, EPBD, and EPLBD were performed in 58 (57%), 20 (20%), and 11 patients (11%), respectively. Endoscopic mechanical lithotripsy was performed in eight patients. Intraoperative bleeding in 24 patients (24%), with bleeding resolving spontaneously in 13 cases (13%). For the remaining 11 patients (11%), endoscopic hemostasis was performed using a spray of epinephrine‐added saline solution in nine patients (8.8%) and stone extraction balloon compression in two patients (2.0%) (Table 3). Hemostasis was successfully achieved in all the patients with intraoperative bleeding.

**TABLE 3:** Outcomes and adverse events of endoscopic retrograde cholangiopancreatography (ERCP) in patients undergoing dialysis.

*n*	102
Naïve papilla, *n* (%)	89 (87)
Periampullary diverticulum, *n* (%)	24 (24)
Largest diameter of the stone(mm), Mean ± SD	6.3 ± 2.9
Number of stones, *n* (%)	
1	44 (43)
≥2	58 (57)
Complete stone removal, *n* (%)	98 (96)
Papillary procedure, *n* (%)	
EST	58 (57)
EPBD	20 (20)
EST + EPLBD	11 (11)
Intraoperative bleeding, *n* (%)	24 (24)
Method of hemostasis, *n* (%)	
Spontaneous hemostatic	13 (13)
Epinephrine‐added saline spray	9 (8.8)
Stone extraction balloon compression	2 (2.0)
Mechanical lithotripsy, *n* (%)	8 (7.8)
Adverse events, *n* (%)	23 (23)

Abbreviations: EPBD, endoscopic papillary balloon dilation; EPLBD, endoscopic papillary large balloon dilation; EST, endoscopic sphincterotomy.

**FIGURE 2:**
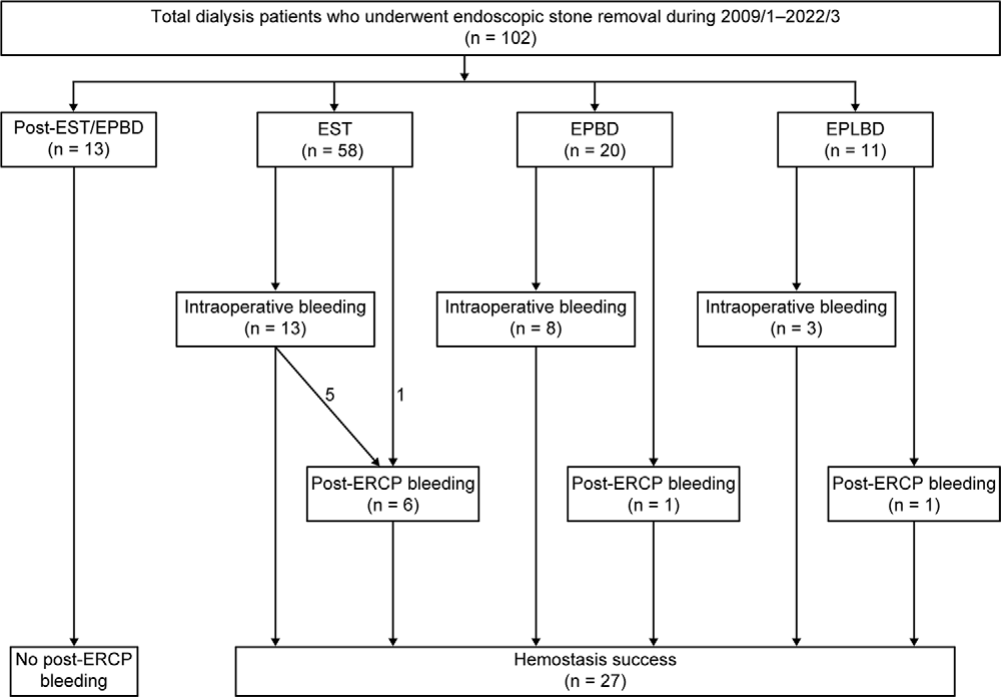
Number of post‐endoscopic retrograde cholangiopancreatography bleeding by the type of papilla procedure. Abbreviations: EST, endoscopic sphincterotomy; EPBD, endoscopic papillary balloon dilation; EPLBD, endoscopic papillary large balloon dilation.

AEs were observed in 23 patients (23%), including post‐ERCP bleeding in eight patients (8%), acute pancreatitis in nine patients (9%), cholangitis in seven patients (7%), hyperamylasemia in one patient (1%), and elevated liver enzymes in one patient (1%) (Table 4). No perforations were observed. The severity of post‐ERCP bleeding was classified as mild in seven cases and moderate in one case. One patient developed moderate bleeding requiring blood transfusion and prolonged hospitalization; however, surgery or interventional radiology was not required. Hemostasis was achieved spontaneously in two cases, and endoscopic hemostasis was successfully performed in the remaining six cases. Among the eight patients with post‐ERCP bleeding, four had received only epinephrine‐containing saline spray for intraoperative bleeding. Figure 2 summarizes the distribution of intraoperative and post‐ERCP bleeding according to the type of papillary intervention.

**TABLE 4:** Number and severity of adverse events.

*n*	102
Acute pancreatitis, *n* (%)	
Mild	6 (5.9)
Moderate	3 (2.9)
Severe	0 (0)
Post‐ERCP bleeding, *n* (%)	
Mild	7 (6.8)
Moderate	1 (1.0)
Severe	0 (0)
Method of hemostasis, *n* (%)	
Thermal coagulation	3 (2.9)
Spontaneous hemostasis	2 (1.9)
Stone extraction balloon compression	1 (1.0)
HSE injection ^a^	1 (1.0)
Clipping	1 (1.0)
Cholangitis, *n* (%)	
Mild	3 (2.9)
Moderate	4 (3.9)
Severe	0 (0)
Perforation, *n* (%)	0 (0)
Hyperamylasemia, *n* (%)	1 (1.0)
Elevated liver enzyme, *n* (%)	1 (1.0)

Abbreviations: ERCP, endoscopic retrograde cholangiopancreatography; HSE, hypertonic saline epinephrine injection.

*Note*: Some patients experienced more than one adverse event.

^a^ One case received an additional self‐assembling hemostatic gel following HSE injection.

### 3.4 Risk of Post‐ERCP Bleeding

Univariate logistic regression analyses showed associations between post‐ERCP bleeding and intraoperative bleeding, continued aspirin use, and naïve papilla. Multivariate analysis identified continued aspirin use (*p* = 0.012) and intraoperative bleeding (*p* = 0.015) as independent factors associated with post‐ERCP bleeding (Table 5). Because no post‐ERCP bleeding events occurred among patients with a non‐naïve papilla, papillary status could not be included in the multivariate model.

**TABLE 5:** Multivariate analysis of factors for post‐endoscopic retrograde cholangiopancreatography (post‐ERCP) bleeding.

	Bleeding (*n* = 8)	No bleeding (*n* = 94)	Univariate analysis *p*‐value	Odds ratio	Multivariate analysis 95% CI	*p*‐Value
Sex (male)	5 (63%)	60 (64%)	0.94			
Age (years)	79.6 ± 5.9	76.4 ± 8.7	0.30			
Platelet count < 10 (×10^4^/µL)	1 (13%)	13 (14%)	0.92			
PT‐INR	1.06 ± 0.06	1.13 ± 0.35	0.59			
Preprocedural cholangitis	3 (38%)	32 (34%)	0.86			
Aspirin continued	3 (38%)	11 (12%)	0.04	21.6	2.0–238.3	0.012
Dialysis period (>60 months)	3 (38%)	48 (51%)	0.46			
Heparin used during dialysis	4 (50%)	41 (44%)	0.73			
Naïve papillae	8 (100%)	81 (86%)	0.04	NE	NE	NE
Stone size >10 mm	1 (13%)	11 (12%)	0.95			
EST performed	6 (75%)	52 (55%)	0.27			
EST + EPLBD performed	1 (13%)	10 (11%)	0.87			
Intraoperative bleeding	5 (63%)	19 (20%)	0.01	15.4	1.7–141.2	0.015

Papillary status was excluded from the multivariate analysis because no bleeding occurred in patients with a non‐naïve papilla.

NE: Not estimable because no events occurred in patients with non‐naïve papillae.

### 3.5 Subgroup Analysis in Patients with a Naïve Papilla

In the subgroup analysis restricted to patients with a naïve papilla, univariate analysis showed that intraoperative bleeding and continued aspirin use were associated with post‐ERCP bleeding (Table S1).

## 4 Discussion

This multicenter retrospective study examined the incidence of AEs and factors associated with post‐ERCP bleeding in dialysis patients who underwent endoscopic stone removal. Post‐ERCP bleeding occurred in eight of 102 patients (8%). Univariate analysis demonstrated associations between post‐ERCP bleeding and a naïve papilla, continued aspirin use, and intraoperative bleeding, whereas multivariate analysis identified continued aspirin use and intraoperative bleeding as factors independently associated with post‐ERCP bleeding. In contrast, continued aspirin use was not significantly associated with intraoperative bleeding in the univariate analysis. These findings suggest that careful periprocedural bleeding management is required in dialysis patients undergoing ERCP. The clinical relevance of the present study lies in its multicenter evaluation of post‐ERCP bleeding after endoscopic stone removal across multiple papillary intervention strategies in dialysis patients, with particular attention to periprocedural antithrombotic management and continued aspirin use.

Previous studies, including those by Nakaji et al., have reported a relatively high incidence of post‐ERCP bleeding in dialysis patients. However, evidence regarding periprocedural antithrombotic management in this population remains limited. These studies also identified anticoagulant therapy as a risk factor for post‐EST bleeding in hemodialysis patients. In contrast to previous studies that identified anticoagulant therapy as a risk factor for post‐EST bleeding in hemodialysis patients, the present study found that continued aspirin use, rather than anticoagulant use, was independently associated with post‐ERCP bleeding. This discrepancy may be partly explained by differences in study populations, target procedures, and periprocedural antithrombotic management.

Recent guidelines generally recommend continuation of aspirin even for high‐bleeding‐risk endoscopic procedures to reduce thromboembolic risk. Therefore, our findings are clinically important because they suggest that dialysis patients may remain at increased risk of post‐ERCP bleeding despite guideline‐based aspirin continuation. Careful periprocedural risk assessment and hemostatic management may therefore be particularly important in dialysis patients receiving aspirin therapy.

In the subgroup analysis restricted to patients with a naïve papilla, post‐ERCP bleeding occurred exclusively in this population, and the associations with intraoperative bleeding and continued aspirin use were consistent with those observed in the overall cohort. These findings may reflect the influence of papillary intervention during initial ERCP procedures in patients with a naïve papilla. Patients with a naïve papilla were more likely to undergo EST or EST combined with EPLBD during stone extraction, whereas patients with a previously treated papilla were more often managed with balloon dilation alone. Therefore, the association between a naïve papilla and post‐ERCP bleeding may reflect the greater use of higher‐bleeding‐risk papillary interventions, particularly EST‐based procedures, rather than papillary status itself.

Intraoperative bleeding has previously been reported to be an independent risk factor for post‐ERCP bleeding in dialysis patients [13, 18]. Consistent with these findings, the present study also demonstrated an association between intraoperative bleeding and post‐ERCP bleeding. In the present study, endoscopic hemostasis was achieved in all cases of intraoperative bleeding, and no patients required interventional radiology or surgery. Because wound healing is impaired in dialysis patients, rebleeding may occur despite successful initial hemostasis. Therefore, careful post‐procedural monitoring is warranted, particularly during the first 5 days after the procedure.

Notably, among the eight patients with post‐ERCP bleeding, five patients had complicated intraoperative bleeding, and four patients were treated with only epinephrine‐added saline solution. Some of these patients experienced rebleeding, indicating that more definitive hemostatic methods may be preferable for managing intraoperative bleeding in dialysis patients. Furthermore, various treatment options for intraoperative bleeding include epinephrine‐added saline solution, HSE injection, stone extraction balloon compression, clipping, and thermal coagulation. In cases of refractory bleeding, the placement of a metallic stent could be considered. Moreover, a novel self‐assembling hemostatic gel has been reported to be useful for post‐EST bleeding and could serve as an alternative when conventional methods are insufficient [22, 23]. Efforts should also focus on preventing intraoperative bleeding. Accordingly, perioperative management of antithrombotic drugs and the selection of papillary procedures should be carefully considered to avoid post‐ERCP bleeding.

The perioperative management of aspirin remains controversial. Current Japanese and international guidelines generally recommend continuing aspirin, even for high‐risk endoscopic procedures such as EST, to reduce the risk of thromboembolic events [20, 24].

Despite these recommendations, continued aspirin use was independently associated with post‐ERCP bleeding in the present study. This suggests that caution should be exercised when performing papillary procedures in dialysis patients on aspirin therapy.

Previous studies have indicated a lower bleeding rate with EPBD compared to EST in dialysis patients [10, 17]. Although EST was not identified as a significant risk factor for bleeding in this study, careful attention to the risk of post‐ERCP bleeding is warranted in dialysis patients who continue aspirin therapy, regardless of whether EST or EPBD is performed. Additionally, recent reports on the efficacy of minimal EST with balloon dilation in dialysis patients suggest that further validation of this technique is warranted [25]. However, minimal EST with balloon dilation was not performed during the current study period, and further investigation is warranted.

Although the risk of aspirin withdrawal should be carefully considered, hemostasis was achieved in all bleeding cases, and no severe bleeding occurred in this study. Because most bleeding events were mild to moderate, decisions regarding antithrombotic withdrawal should be individualized after carefully balancing the risks of bleeding and thrombosis. Particular attention should be paid to intraoperative bleeding in dialysis patients who continue aspirin therapy, as more aggressive hemostatic management may be required in this population. These findings underscore the importance of careful risk stratification and meticulous hemostatic management during ERCP in patients undergoing hemodialysis.

The mechanism underlying this association remains unclear. Delayed mucosal healing, uremia‐related platelet dysfunction, and repeated anticoagulation during hemodialysis may contribute to delayed, rather than immediate, post‐ERCP bleeding.

This study has several limitations. Its retrospective design, small sample size, and limited number of bleeding even0074s may affect the robustness of the conclusions. The selection of papillary interventions and perioperative antithrombotic management varied across institutions, and procedure time was not consistently recorded; therefore, procedure time and cannulation difficulty could not be evaluated. In addition, the definition of intraoperative bleeding was operator‐dependent, and minor bleeding events may have been inconsistently documented because of the retrospective multicenter design. Furthermore, ERCP procedures were performed by endoscopists with varying levels of experience across institutions, and the indications for urgent or semi‐urgent ERCP may also have differed among centers. The study was not prospectively powered for multivariable analysis, potentially resulting in imprecise estimates. Finally, the absence of post‐ERCP bleeding in patients with a non‐naïve papilla and the lack of a non‐dialysis control group precluded robust subgroup comparisons and direct assessment of dialysis‐related bleeding risk.

In conclusion, intraoperative bleeding and continued aspirin use were independently associated with post‐ERCP bleeding in dialysis patients who underwent stone removal. These findings underscore the need for careful management of bleeding risk in patients undergoing dialysis. Future studies are needed to validate these results and refine management strategies for dialysis patients undergoing ERCP.

## Author Contributions

**Takashi Kaneko** was involved in study design and data interpretation. **Masaki Nishimura** wrote the manuscript. **Haruo Miwa** contributed to the analysis and interpretation of data and writing – review and editing. All authors reviewed the manuscript draft and revised it critically for intellectual content. All authors approved the final version of the manuscript for publication.

## Funding

The authors have nothing to report.

## Ethics Statement

The study protocol was approved by the Institutional Review Board of Yokohama City University and adhered to the provisions of the Declaration of Helsinki (revised in Fortaleza, Brazil in October 2013). The IRB approval number is B190300046.

## Consent

Written informed consent for ERCP was obtained from all patients. Because of its retrospective design, the requirement for written informed consent for study participation was waived, and an opt‐out approach using medical records was applied. The study was approved by the appropriate institutional review boards or ethics committees at all participating centers.

## Conflicts of Interest

The authors declare no conflicts of interest.

## Supporting information




**TABLE S1** Subgroup analysis in patients with a naïve papilla.

## Data Availability

All data generated or analyzed during this study are included in this published article and its Supporting Information.
