# Supplementary material for: Post‐Endoscopic Retrograde Cholangiopancreatography Bleeding After Endoscopic Stone Removal in Dialysis Patients: A Multicenter Retrospective Study
Source: DEN Open. 2026 Aug 7;7(1):e70395. doi: 10.1002/deo2.70395 (PMC13449692; doi:10.1002/deo2.70395)
Supplement: Supplementary file 1 [file DEO2-7-e70395-s001.docx]

**Table S1.**

|  | **Bleeding (n = 8)** | **No bleeding (n = 81)** | **Univariate analysis *p*-value** |
| --- | --- | --- | --- |
| Sex (male) | 5 (63%) | 52 (64%) | 0.92 |
| Age (years) | 79.6 ± 5.9 | 76.2 ± 8.2 | 0.16 |
| Platelet count < 10 (×10^4^/μL) | 1 (13%) | 11 (14%) | 0.93 |
| PT-INR | 1.06 ± 0.06 | 1.14 ± 0.37 | 0.10 |
| Aspirin continued | 3 (38%) | 6 (7.4%) | 0.03 |
| Dialysis period (>60 months) | 3 (38%) | 45 (56%) | 0.33 |
| Heparin used during dialysis | 5 (63%) | 37 (46%) | 0.36 |
| Stone size >10mm | 1(13%) | 10(12%) | 0.99 |
| EST performed | 6 (75%) | 52 (64%) | 0.53 |
| EST+EPLBD performed | 1 (13%) | 10 (12%) | 0.99 |
| Intraoperative bleeding | 5 (63%) | 19 (23%) | 0.03 |
